# One-Shot Resin 3D-Printed Stators for Low-Cost Fabrication of Magic-Angle Spinning NMR Probeheads

**DOI:** 10.1021/acs.analchem.3c01323

**Published:** 2023-06-28

**Authors:** Daniel Pereira, Mariana Sardo, Ildefonso Marín-Montesinos, Luís Mafra

**Affiliations:** CICECO—Aveiro Institute of Materials, Department of Chemistry, University of Aveiro, 3810-193 Aveiro, Portugal

## Abstract

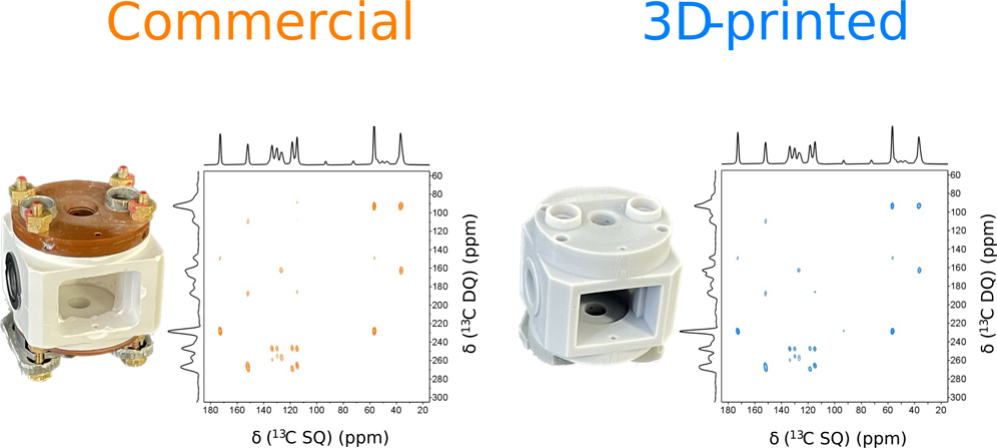

Additive manufacturing such as three-dimensional (3D)-printing has revolutionized the fast and low-cost fabrication of otherwise expensive NMR parts. High-resolution solid-state NMR spectroscopy demands rotating the sample at a specific angle (54.74°) inside a pneumatic turbine, which must be designed to achieve stable and high spinning speeds without mechanical friction. Moreover, instability of the sample rotation often leads to crashes, resulting in costly repairs. Producing these intricate parts requires traditional machining, which is time-consuming, costly, and relies on specialized labor. Herein, we show that 3D-printing can be used to fabricate the sample holder housing (stator) in one shot, while the radiofrequency (RF) solenoid was constructed using conventional materials available in electronics stores. The 3D-printed stator, equipped with a homemade RF coil, showed remarkable spinning stability, yielding high-quality NMR data. At a cost below 5 €, the 3D-printed stator represents a cost reduction of over 99% compared to repaired commercial stators, illustrating the potential of 3D-printing for mass-producing affordable magic-angle spinning stators.

## Introduction

Solid-state nuclear magnetic resonance (ssNMR) spectroscopy is an essential analytical technique for the characterization of the structure and dynamics of powdered samples encompassing small molecules,^1−3^ biomolecules,^4−7^ and materials.^7−9^ NMR spectra of solids typically yield very broad resonances due to the absence of fast molecular tumbling (as typically observed in solution NMR), thus precluding a more detailed analysis of the NMR spectra. High-resolution spectra are usually obtained by rotating the sample at the magic angle.^10^ Under this condition, anisotropic interactions are averaged out thus leading to line-narrowing.

Magic-angle spinning (MAS) is now a routine technique in the field of ssNMR. In practice, the solid sample is packed inside a cylindrical sample holder (with diameter ranging from 9.0 to 0.5 mm),^11,12^ known as a rotor, and then placed inside the stator, where the rotor is frictionlessly spun at MAS frequencies from 5 kHz^11^ up to 170 kHz.^13^ The stator is a complex part comprising two crucial pneumatic systems, namely, bearing jets and drive jets, which are used simultaneously to spin the rotor.^14−17^ The bearing jets are used to lubricate the rotor with gas, while the drive jets are employed to induce rotational motion to the rotor.^15^ MAS NMR experiments often require long periods of stable spinning. For instance, some NMR pulse sequences need rotor synchronization,^18^ which demands highly stable spinning frequencies to work efficiently. In other cases, disastrous events such as rotor crashes may take place derived from spinning instabilities. A rotor crash results in the destruction of crucial elements, *e.g.*, rotor (and sample), stator, radiofrequency (RF) solenoid coil, or the simultaneous damage thereof. In addition to the financial hurdle resulting from a rotor crash (*e.g.*, probe transportation, repair costs), there is a cascade of events that affects the daily routine of NMR labs (*e.g.*, logistic complications due to experimental delays).

Recently, some groups have explored the use of additive manufacturing methods (three-dimensional printing (3D-printing)) for fast and cost-efficient fabrication of NMR components for distinct applications, from solution to ssNMR.^19−24^ Günne’s lab reported the fabrication of double bearing stators for 7.0, 4.0, and 3.5 mm pencil-style rotors using filament-based 3D-printing technology,^23^ showing that satisfactory rotational speeds and stabilities could be achieved. Recently, Griffin’s group published a study wherein a 3D-printed stator for 3.2 mm rotors was built using a combination of 3D-printing and traditional machining methods.^24^ However, the most important components of this turbine system were produced by traditional machining methods (*e.g.*, radial air bearings, stator counterbores for the radial bearings, axial bearing plate, and Bernoulli valve) and specialized 3D-printing services for the fabrication of the stator body and drive plate.

Most rotor crashes lead to irreparably deformed RF solenoid coils. Therefore, it is important to introduce methods that enable the fabrication of in-house transceiver coils. Despite the numerous theoretical works published for RF coil design and optimization,^25−29^ publications describing practical methods for their in-house production are scarce. 3D-printing technology has also been successfully applied for the smart design of RF transceiver coils. Recently, a novel method has been introduced to wind high-performance and homogeneous variable-pitch RF transceiver solenoids by using dissolvable 3D-printed templates.^30^ This method opens the door for NMR labs to build high-performing RF transceiver coils at very reduced prices while maintaining sensitivity and homogeneity.

Herein, we describe a method for in-house fabrication of a one-shot resin 3D-printed 4.0 mm MAS stator for ssNMR spectroscopy, using stereolithography (SLA) 3D-printing technology. Additionally, we produced a homemade RF solenoid coil using an easy-to-implement method. To demonstrate the robustness of the probehead, we tested the 3D-printed stator and RF solenoid coils on a model sample by performing rotor-synchronized NMR experiments. We compared the cost and performance of our 3D-printed stator/coil with commercially available solutions and found that our methodology provides an overall price reduction higher than 99%.

## Materials and Methods

### Stator and Coil Alignment Tool Design

The 4.0 mm MAS stator and the coil alignment tool were designed using Fusion 360 (Autodesk, CA) software. The stator was modeled by reverse engineering a 4.0 mm Bruker stator. The key parts for stator, *i.e.*, radial and axial bearings, drive ring, and Bernoulli valve, were adapted from the Griffin’s group design.^24^ The coil alignment tool was designed based on the 4.0 mm stator and used to verify the proper position of the RF coil with respect to the rotor spinning axis within the stator.

### Coil Fabrication

For the coil fabrication, a regular copper wire with 0.8 mm diameter was used. A brass wire with 0.4 mm diameter was also used to ensure the correct spacing between solenoid turns (1/2 of copper wire diameter) (32, 36). The copper and brass wires were simultaneously hand-wrapped on a 4.5 mm drill (Supporting Figure 1). The RF coil was built with an inner diameter of 4.5 mm, 7 turns, and a height of 9.2 mm, and aligned inside the stator with the aid of the coil alignment tool. Finally, the RF coil was secured in the coil block with super glue. All of the materials used for the fabrication of the coil (∼30 m of copper and brass wire and the drill as observed in Supporting Figure 1) were bought from conventional hardware/tool stores.

### 3D-Printing and Postprocessing Methods

All parts were printed with an Anycubic Photon M3 Plus (Anycubic, Shenzen, CN) SLA printer equipped with a 6k monochrome liquid-crystal display (LCD) yielding an XY resolution of 34 μm using Anycubic Craftsman resin. The slicing of the models was done with CHITUBOX software. The full stator was printed in less than 2 h and, after postprocessing, it was assembled in the probehead.

To obtain high-quality prints with smooth surfaces, appropriate postprocessing treatments were applied. First, the parts were washed in an isopropanol bath to remove any uncured resin. Finally, the parts were exhaustively dried and cured under ultraviolet (UV) light for 10–30 min.

### Solid-State NMR Experiments

**Figure 1:**
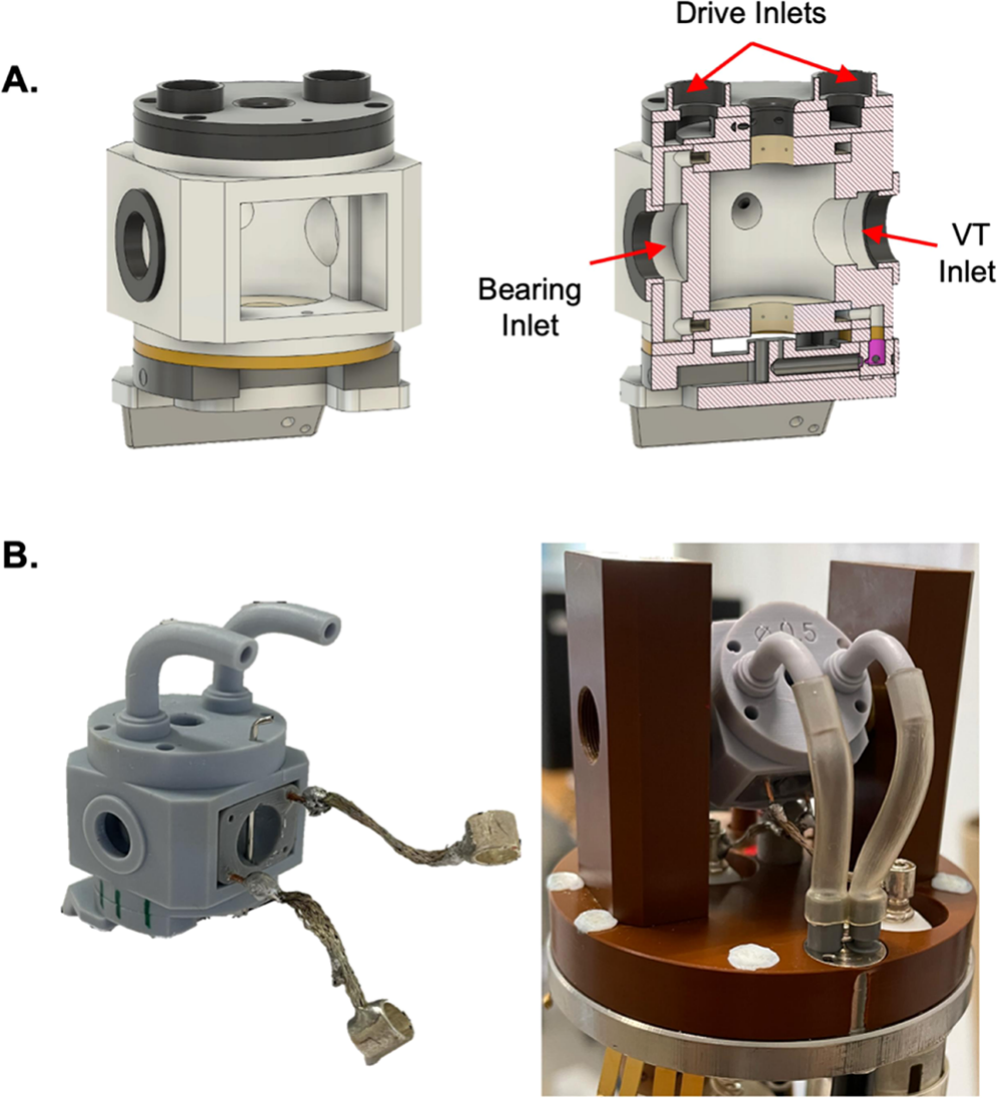
4.0 mm MAS stator: (A) Computer-assisted design (CAD) model (left) and sectional view of the 4.0 mm stator, highlighting the variable temperature (VT), bearing, and gas inlets (right); (B) 3D-printed 4.0 mm stator equipped with a homemade RF coil (left) and 3D-printed 4.0 mm stator retrofitted into the probehead (right).

^1^H, ^13^C, and ^15^N NMR spectra were acquired on a Bruker Avance III 400 spectrometer operating at a B_0_ field of 9.4 T, with ^1^H/^13^C/^15^N Larmor frequencies of 400.13/100.61/40.56 MHz, respectively. The experiments were performed on a double-resonance 4.0 mm Bruker MAS probe retrofitted with in-house 3D-printed stator and RF coil as shown in Figure 1. A 4.0 mm Bruker triple-resonance MAS probe was also used for the sake of comparison. The samples were packed into ZrO_2_ rotors with Kel-F caps employing spinning rates of 4.0–8.0 kHz. The magic angle of the 3D-printed stator was adjusted with KBr (Supporting Figure 2) upon installation of the RF coil. ^13^C chemical shifts are quoted in ppm from α-glycine (secondary reference, C=O at 176.50 ppm). The ^13^C CPMAS spectra were acquired under the following experimental conditions: ^1^H 90° pulse set to 3.0 μs corresponding to a radiofrequency (RF) field of ∼83.3 kHz; the CP step was performed with a contact time (CT) of 3000 μs using a 50–100% RAMP shape pulse on the ^1^H channel and a 50 kHz square pulse on the ^13^C channel; recycle delay of 3 s. During the acquisition, a SPINAL-64 decoupling scheme^31^ was employed using a pulse length for each basic decoupling unit of 5.0 μs, corresponding to an RF field of ∼91.7 kHz. The ^13^C–^13^C correlation experiment was accomplished by using the POST-C7 pulse sequence for dipolar recoupling.^32^ The two-dimensional (2D) ^13^C–^13^C double-quantum/single-quantum (DQ/SQ) POST-C7 spectra were acquired using seven post-C7 blocks for the DQ excitation and reconversion times (12.25 ms) employing a ^13^C RF field of 56 kHz. Continuous wave proton decoupling of ∼83.3 kHz was applied during ^13^C recoupling. Heteronuclear decoupling during the acquisition time and CP steps used the same experimental conditions as referred previously. The ^15^N CPMAS spectrum (Supporting Figure 3) was acquired using the same CP experimental conditions as mentioned above. The CP contact time was 8000 μs. ^15^N chemical shifts are quoted in ppm from nitromethane.

## Results

### 3D-Printed NMR Stator: Spinning Tests and Stability Analysis

The 3D-printed 4.0 mm MAS stator described in this work (Figure 1) is a double bearing type of turbine, comprising two sets of radial bearing jets, one axial bearing jet and one set of drive jets, as shown in Figure 1A. The radial and axial bearing jets were designed with a nozzle diameter of 0.5 and 0.6 mm, respectively. The drive ring geometry was designed based on previously proposed models using five jets at a quasi-tangential orientation with respect to the rotor cap fins.^14,15^Figure 1B depicts the postprocessed 3D-printed stator that was subsequently assembled into the probehead.

**Figure 2:**
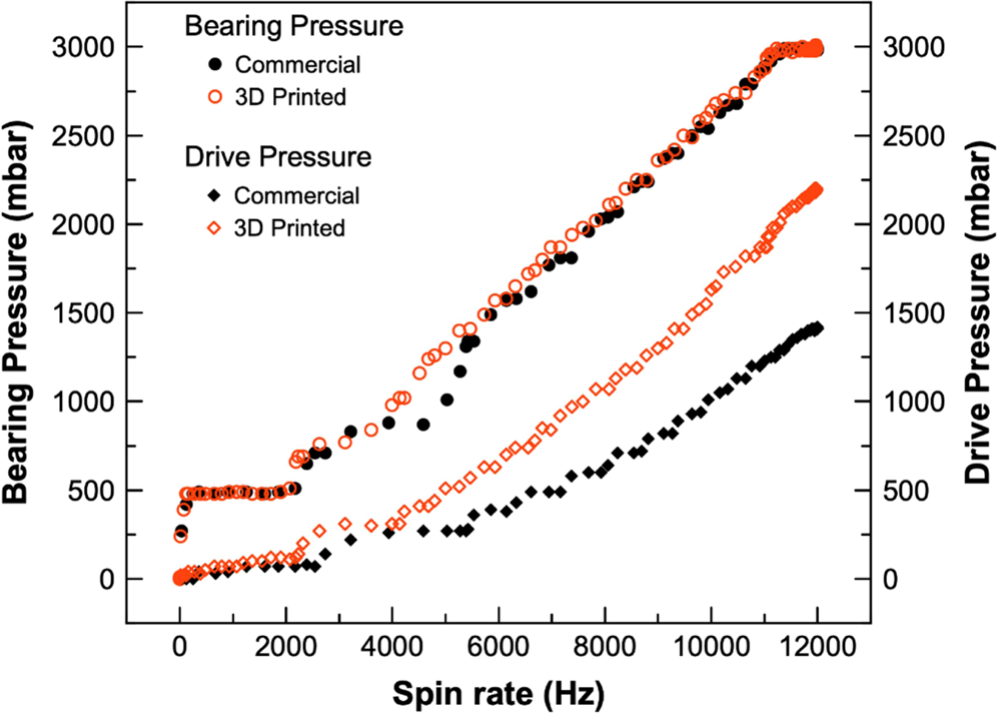
MAS pneumatic spinning requirements. The bearing and drive gas pressures were measured as a function of the spin rate in both 3D-printed and commercial versions of the stator. The nominal spin rate was set to 12.0 kHz, and the rotor was spun in automatic mode, with bearing and drive pressures controlled by a MAS-2 Bruker unit. The same rotor, packed with ^13^C/^15^N Tyr·HCl, was used for both stators.

MAS tests were performed in the 4.0 mm 3D-printed stator, mounted on a commercial probe, to assess the spinning pneumatic requirements and stability. The bearing and drive gas pressures and spinning frequencies were recorded as a function of time for both the 3D-printed and commercial versions of the stator (Figure 2). During our tests, the 3D-printed stator reached a maximum of ∼12 kHz. Although the spinning rate dependence on the bearing pressure shows similar trends in both 3D-printed and commercial stators, the drive pressure of the former requires noticeably more drive pressure than the latter to reach the same MAS rate, likely associated with the presence of printing defects affecting the drive nozzles geometry.

Spinning rate stability tests were also performed in both stators by recording the MAS rate over time. Only marginal differences in the spinning rate stability are observed as evaluated from the mean MAS rate and standard deviation values (Table 1).

**Table 1:** Spinning Performance of 3D-Printed and Commercial 4.0 mm Stators^a^

stator	test length (min)	ν_R_, Hz	, Hz	σ_R,_ Hz
3D-printed	1020	8000	7999.006	5.32
commercial	1020	8000	7998.993	5.11

a The spin rate was measured over time using the same rotor packed with ^13^C/^15^N Tyr·HCl in both stators. ν_R_, , and σ_R_ denote the nominal, mean, and standard deviation of the MAS frequency, respectively.

### Homemade RF Coil Performance Evaluation

**Figure 3:**
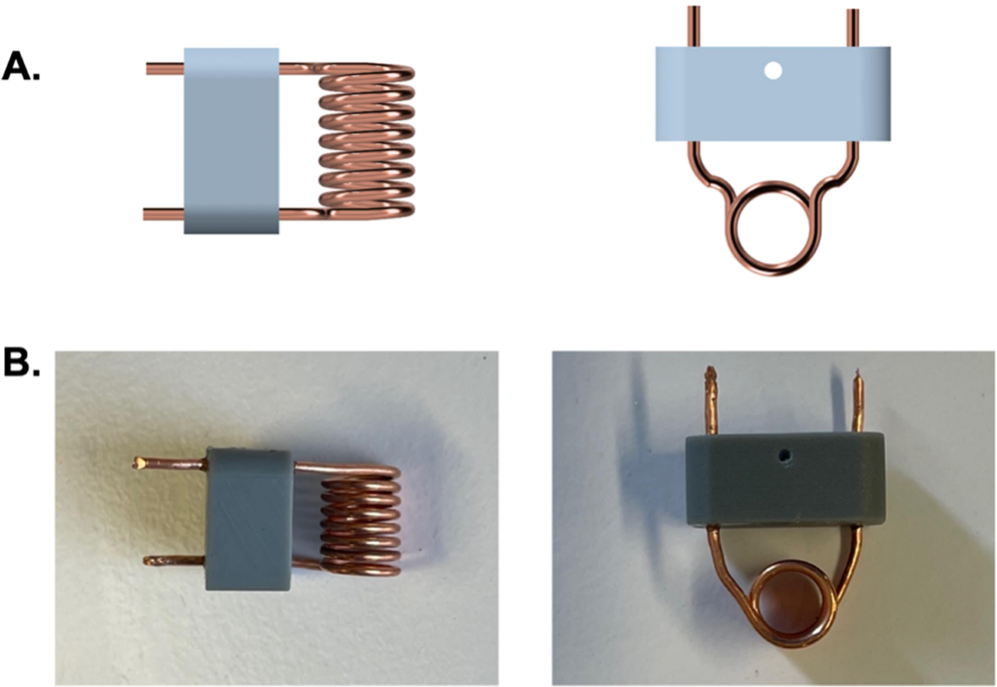
RF solenoid coil. (A) CAD model of a 4.0 mm RF coil; (B) homemade 4.0 mm RF coil.

Figure 3 shows the homemade built RF solenoid coil that has been installed in the 3D-printed stator. NMR coils may have multiple geometries, among which solenoids are likely the best-characterized type of axial resonators, often used in ssNMR.^30,33^ This type of NMR coils is particularly easy to fabricate, presents reasonably good signal-to-noise ratio (SNR) and high filling factors, and is able to yield strong RF magnetic fields (B_1_) when oriented at the magic angle. It is worth mentioning that the discussion ahead compares the performance of our homemade RF coil (installed in the 3D-printed stator) and the commercial probehead.

**Figure 4:**
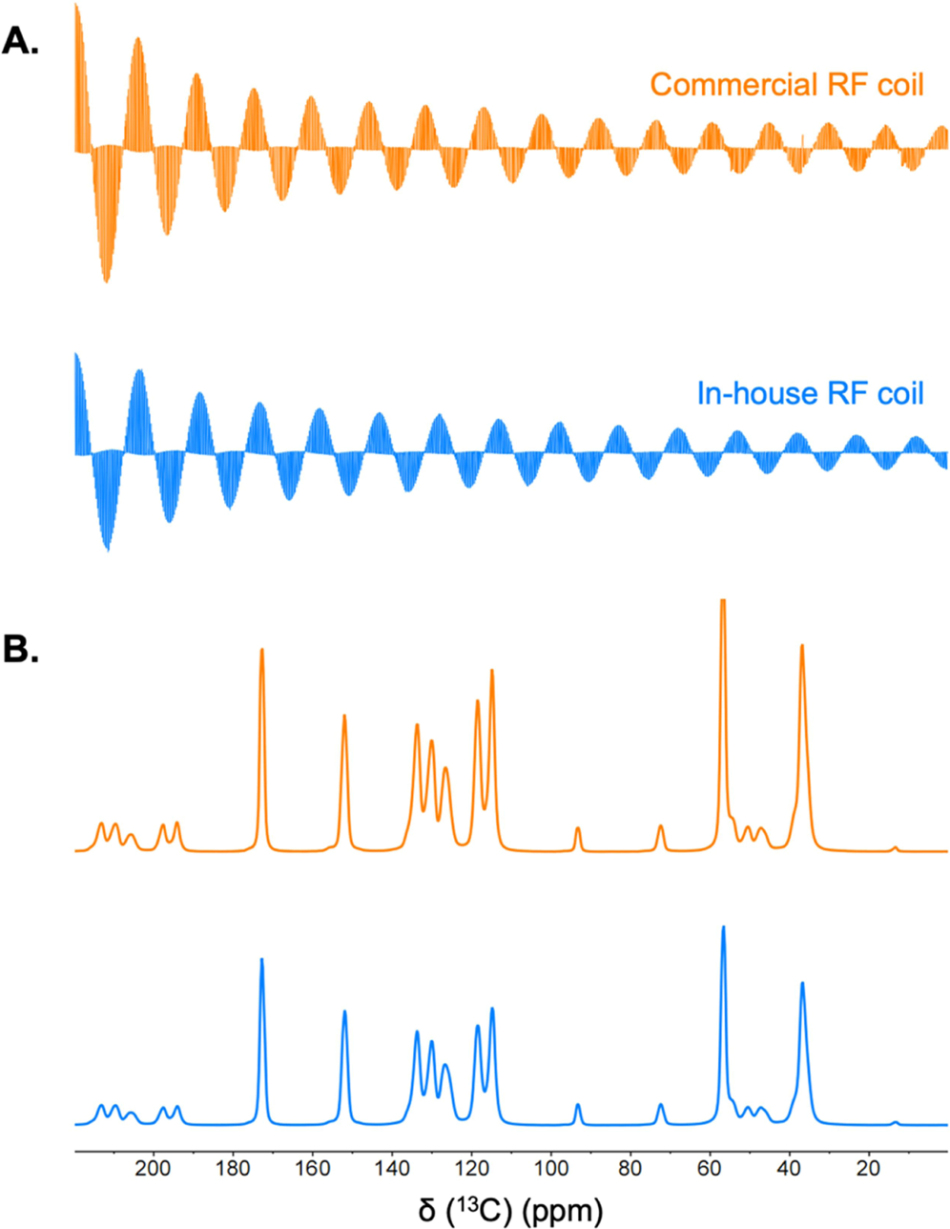
Characterization of the in-house RF solenoid system. NMR spectra of ^13^C/^15^N-enriched Tyr·HCl were recorded at a field strength of 9.4 T and a MAS rate of 8.0 kHz. The 3D-printed stator and RF coil were retrofitted into a double-resonance Bruker MAS probe (blue lines). A commercial 4.0 mm Bruker triple-resonance MAS probe was used for comparison (orange lines). (A) ^13^C RF nutation plot. The irradiation offset was set to the carbonyl peak at 172.8 ppm. (B) ^13^C CPMAS NMR spectra, acquired in the same conditions.

The ^13^C nutation experiment shows that the homemade RF coil has a similar RF B_1_ homogeneity compared to the commercial option (Figure 4A). Despite their similar B_1_ homogeneities, the homemade RF coil exhibits a signal loss of approximately 30% compared to the commercial system (Figure 4B). This signal loss is expected as the homemade solenoid presents a larger inner diameter (4.5 mm) compared to the commercial solenoid (4.4 mm). The larger diameter leads to smaller inductance values and, therefore, a reduced magnitude of the coil filling factor.^34,35^

**Figure 5:**
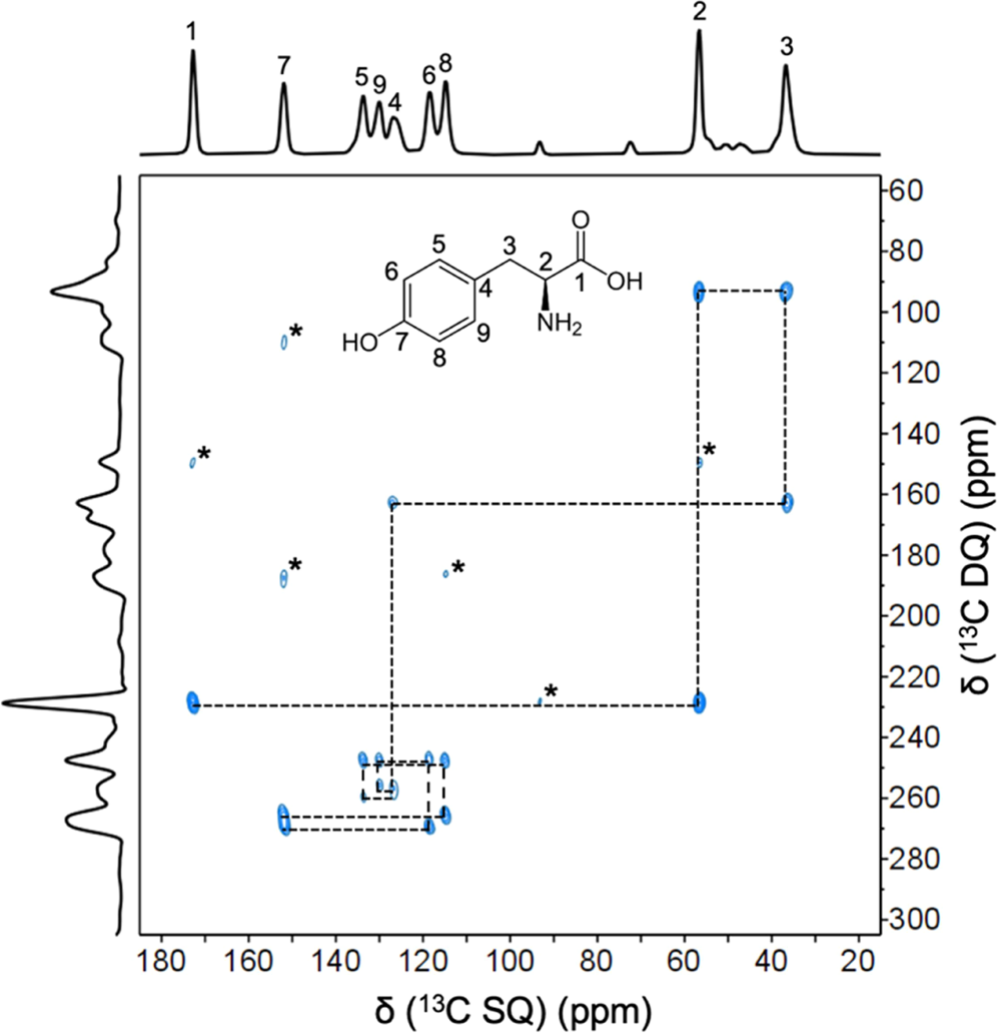
2D ^13^C–^13^C DQ/SQ POST-C7 NMR spectrum of ^13^C/^15^N Tyr·HCl recorded at a field strength of 9.4 T. The spectrum was recorded using a MAS frequency of 8.0 kHz, 512 points were recorded for each t1 increment, and 32 scans per point were used. The recycle delay was set to 3 s. The total experimental time was ∼14 h; * depicts spinning side bands.

The experimental performance of the in-house 3D-printed stator/RF coil combo was further verified by acquiring a 2D ^13^C–^13^C DQ/SQ correlation experiment using the POST-C7 Rotor-synchronized pulse sequence (Figure 5). This experiment requires both exceptional spinning stability and a high number of RF pulses to achieve high-quality spectra. The RF power requirements of this type of pulse sequences often generate thermal fluctuations, which may affect the 3D-printed resin integrity. The recorded 2D NMR spectrum of ^13^C/^15^N-enriched Tyr·HCl (Figure 5) is in good agreement with the spectra of the same compound found in the literature.^36,37^Supporting Figure 4 shows the same 2D spectrum recorded in a commercial probehead. This experiment proves that a 3D-printed stator equipped with a homemade RF coil can be effectively used to perform complex experiments capable of yielding high-quality NMR spectra. Additionally, ^1^H experiments were performed to compare the background signal between a 3D-printed and commercial system (Supporting Figures S5 and S6). The proton background signal, in both systems, is comparable (Supporting Figure 5). Supporting Figure 6 shows the ^1^H spectrum of ^13^C/^15^N-enriched Tyr·HCl acquired using direct-excitation and Hahn Echo experiments, demonstrating that the background signal can be easily suppressed by performing echo experiments.

## Discussion

Traditionally, the field of ssNMR spectroscopy utilizes machined ceramic MAS stators, often made of yttria-stabilized zirconia, due to its favorable properties for constructing the turbines and its ability to be accurately machined into complex parts with high precision. Additionally, these ceramics exhibit a low level of background signal for the most measured nuclides in NMR experiments. The need for machining techniques to produce MAS stators has affected the availability of such components, which are exclusively produced by instrument builders. Excluding a small number of isolated examples of skilled researchers capable of producing their own variations of MAS stators,^13,14,38−40^ the ssNMR community has been highly dependent on commercial instrument providers every time the MAS stator is damaged. Therefore, additive manufacturing emerges as a convenient and cost-efficient method for the rapid in-house fabrication of NMR probehead parts such as MAS stators.

Herein, multiple tests were performed to demonstrate the robustness and capabilities of a 3D-printed stator equipped with a homemade RF coil system, proving that a functional MAS stator/RF coil system can be built using readily available resources. Despite an overall loss of signal in the range of ∼30% observed for the homemade RF coil, it is important to highlight that this comparison is based on results acquired using two distinct 4.0 mm NMR probes (double-resonance probe for the 3D-printed system and triple-resonance probe for commercial system), equipped with distinct electronic components and, therefore, the comparison should not be taken as a definitive 1:1 comparison. Rather, a more direct and careful comparison should be performed by installing both 3D-printed stator/homemade RF coil and commercial stator/RF coil in the same probehead; however, such analysis is beyond the scope of this work.

The 3D-printed stator/homemade RF coil system demonstrates a remarkable cost advantage compared to commercial options, estimated to be less than 5 € (Table 2). The cost may vary based on the types (and purity) of copper wire used for the RF coil and photoresin used for the 3D-printing. Nevertheless, this price fluctuation per stator is insignificant compared to commercial alternatives. For instance, instrument developers usually charge 10–20 k€ for the replacement of a stator/RF coil combo, whereas our results demonstrate a cost reduction of more than 99%, portraying an extremely appealing cost/quality ratio.

**Table 2:** Cost Analysis of 3D-Printed/Homemade Hardware

	parts/equipment	costs, €
homemade	3D printer + wash-cure station	∼1000
	copper and brass wire (∼30 m)^a^	∼10
	resin bottle (1 kg)^b^	∼75
	3D-printed stator	∼1.50
	RF coil	∼0.07
commercial	stator + RF coil	∼10–15 k

a 30 m is equivalent to at least 150 × 4.0 mm coils.

b 1 kg of resin is equivalent to at least 50 × 4.0 mm stators.

The cost of an SLA 3D printer, processing station, and photosensitive resin bundle is in the range of ∼1000 €, a very affordable capital cost for most laboratories. Such a printer may be used to produce other crucial components for MAS NMR spectroscopy (*e.g.*, drive caps^24,41^), in addition to other laboratory applications.^42,43^ Additive manufacturing methods are expected to find applications for smaller rotor sizes, as well as novel stator designs tailored for specific applications.

## Supporting Information Available

Experimental details, and methods, including photographs of the material and methods used for the fabrication of RF coil and ^1^H, ^13^C, ^15^N, and ^79^Br NMR spectra used for the characterization of the 3D-printed and RF coil (PDF)
