# Supplementary material for: One-Shot Resin 3D-Printed Stators for Low-Cost Fabrication of Magic-Angle Spinning NMR Probeheads
Source: Anal Chem. 2023 Jun 28;95(27):10384–9. doi: 10.1021/acs.analchem.3c01323 (PMC10339279; doi:10.1021/acs.analchem.3c01323)
Supplement: Supplementary file 1 — ac3c01323_si_001.pdf [file ac3c01323_si_001.pdf]

Supplementary Information

One-shot resin 3D-printed stators for low-cost fabrication of magic-angle spinning NMR probeheads

Authors
Daniel Pereira, Mariana Sardo, Ildefonso Marín-Montesinos, Luís Mafra*

Affiliations
CICECO – Aveiro Institute of Materials, Department of Chemistry, University of Aveiro, 3810-193 Aveiro, Portugal

*Corresponding author: lmafra@ua.pt (L.M)

Table of contents
Supplementary Figure S1: RF coil fabrication....................S2
Supplementary Figure S2: Magic-Angle Adjustment....................S3
Supplementary Figure S3: ^{15}N spectra of Tyrosine in 3D printed stator with homemade RF coil S4
Supplementary Figure S4: Performance comparison between 3D printed stator with homemade RF coil vs commercial system....................S5
Supplementary Figure S5: ^{1}H background signal of 3D printed and commercial system....................S6
Supplementary Figure S6: ^{1}H spectra of Tyrosine in 3D printed stator with homemade RF coil S7

A.

B.

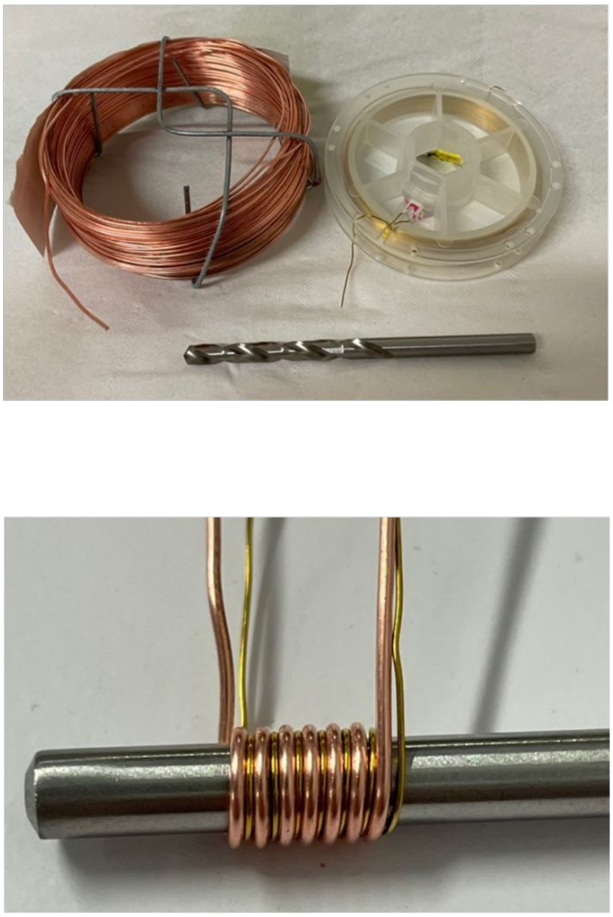

Fig. S1. RF coil fabrication. (**A.**) materials used for the fabrication of the RF solenoid coil; (**B.**) RF coil winded in a 4.5 mm drill. Copper wire and brass wire with a diameter of 0.8 and 0.4 mm, respectively, were used to build the RF solenoid. All the materials used were bought on a conventional hardware/tool stores.

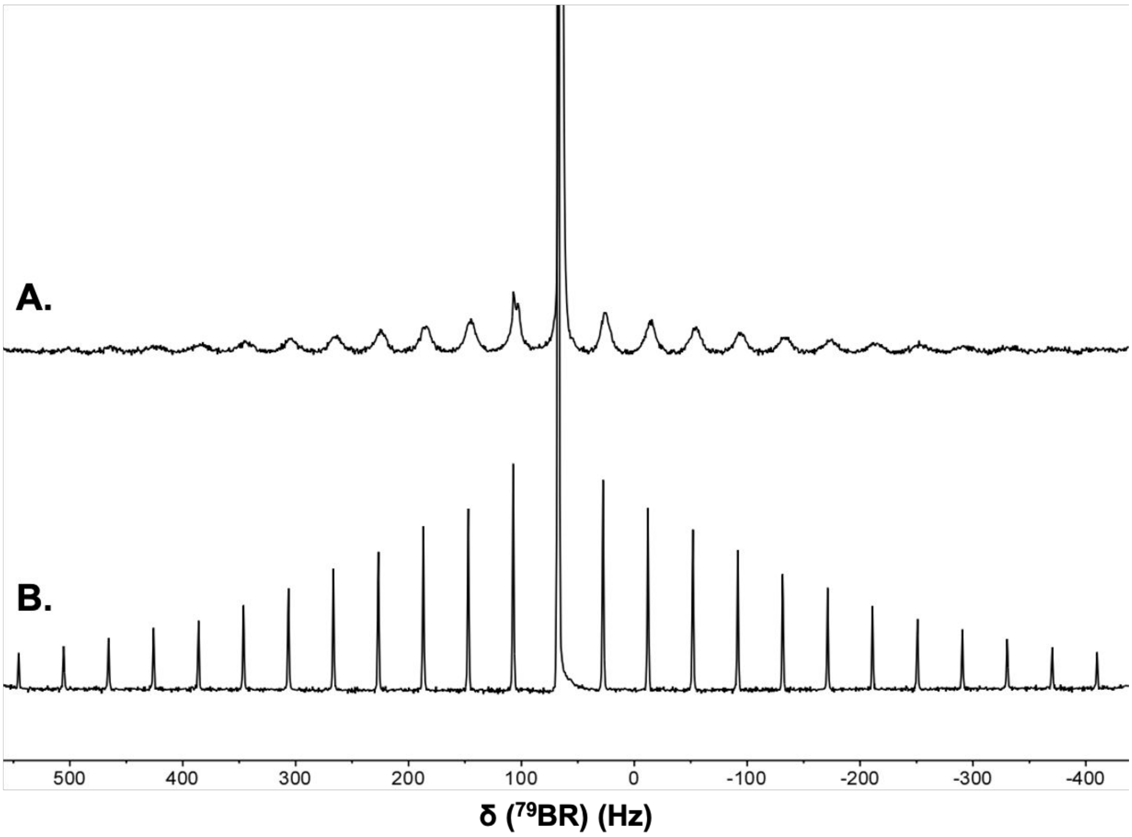

Fig. S2. Magic-angle adjustment. ^{79}Br NMR spectra of KBr recorded at a spinning frequency of 4 kHz. (**A.**) Before Magic-Angle adjustment; (**B.**) After Magic-Angle adjustment.

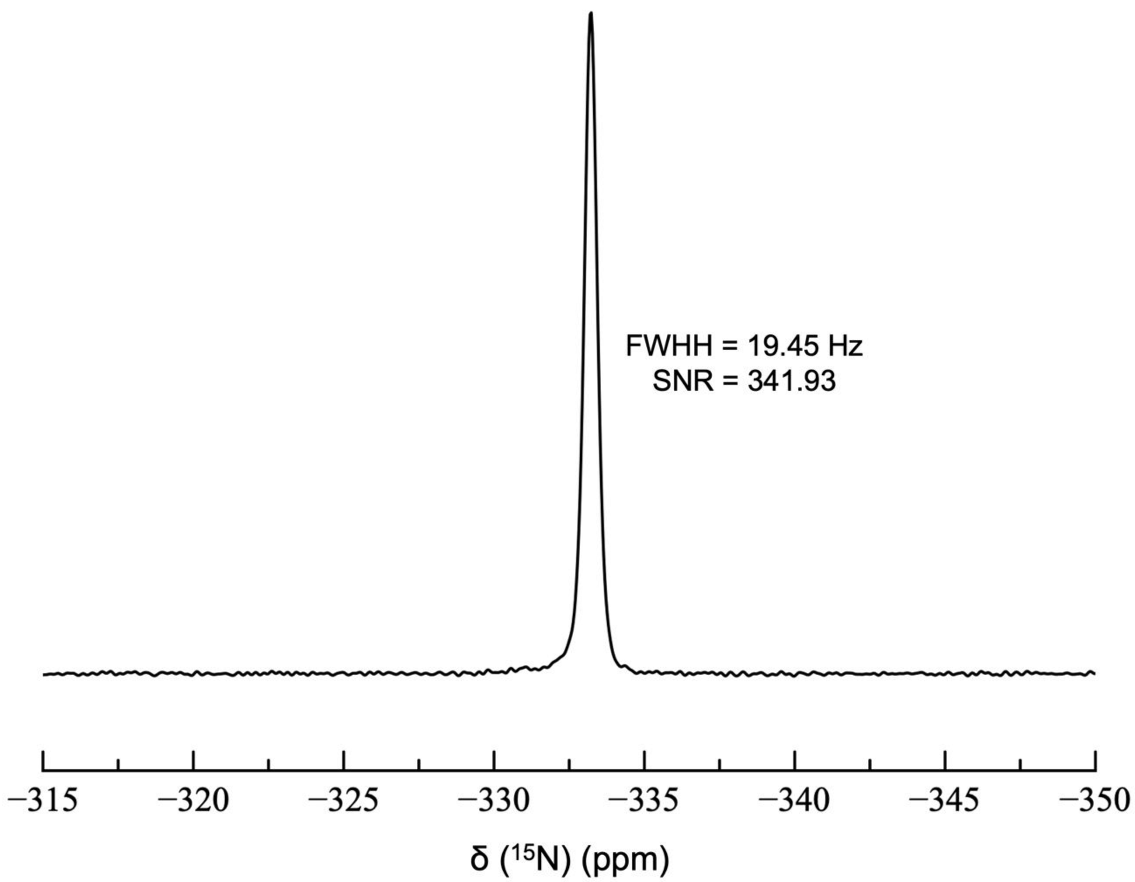

Fig. S3. ^{15}N CP NMR spectra of $^{13}C/^{15}N$-labeled Tyr·HCl recorded at a field strength of 9.4 T. The ^{15}N NMR spectra of Tyr·HCl was recorded at a spinning frequency of 8.0 kHz. The full width at half height (FWHH) and the signal to noise ratio (SNR) for the ^{15}N signal is presented in the spectrum.

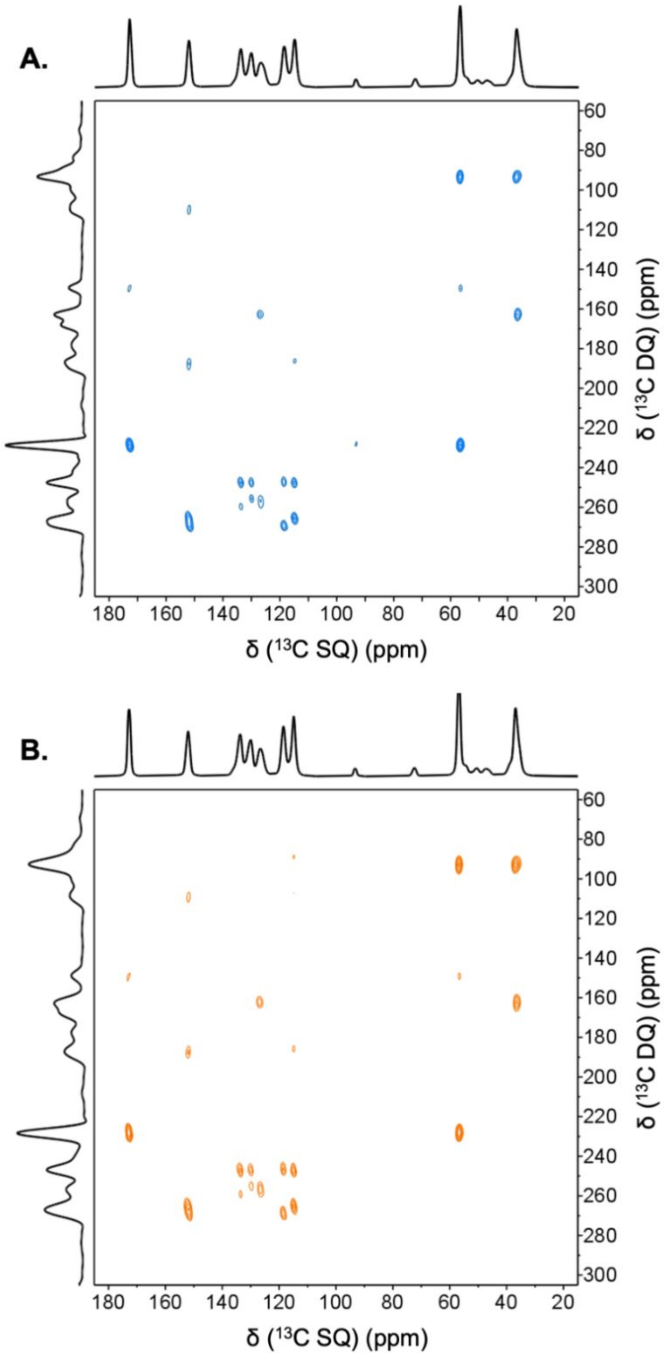

Fig. S4. 2D ^{13}C-^{13}C DQ/SQ POST C7 NMR spectra of $^{13}C/^{15}N$-labeled Tyr·HCl recorded at a field strength of 9.4 T. Each spectrum was recorded using a MAS frequency of 8.0 kHz, 512 t1 increments and 32 repetitions for each t1 increment. The recycle delay was 3 s. ***Spectrum A.*** (blue contours) was recorded with a 3D-printed stator equipped with a homemade RF coil; **Spectrum B.** (orange contours) was recorded with a commercial stator/RF coil.

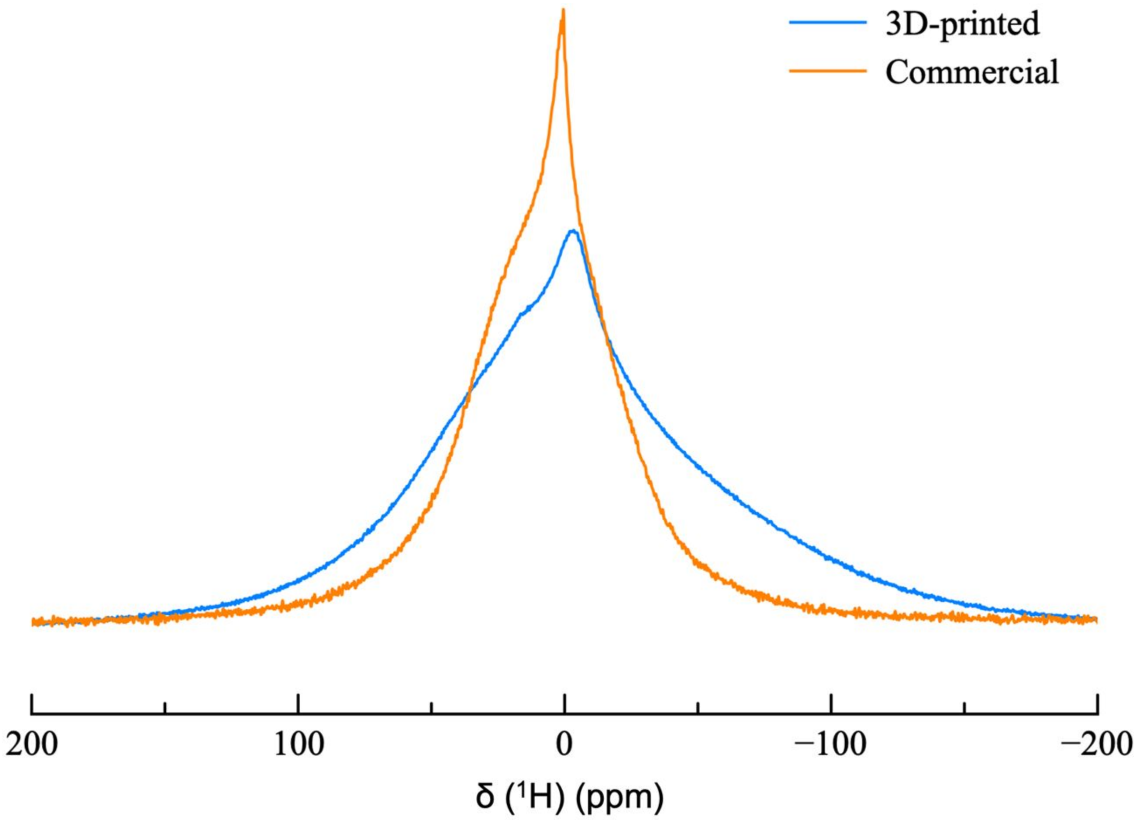

Fig. S5. ^{1}H NMR spectra of probe background signals recorded at a field strength of 9.4 T. The ^{1}H background signal was acquired without rotor, in both commercial and 3D-printed systems, by direct single pulse excitation.

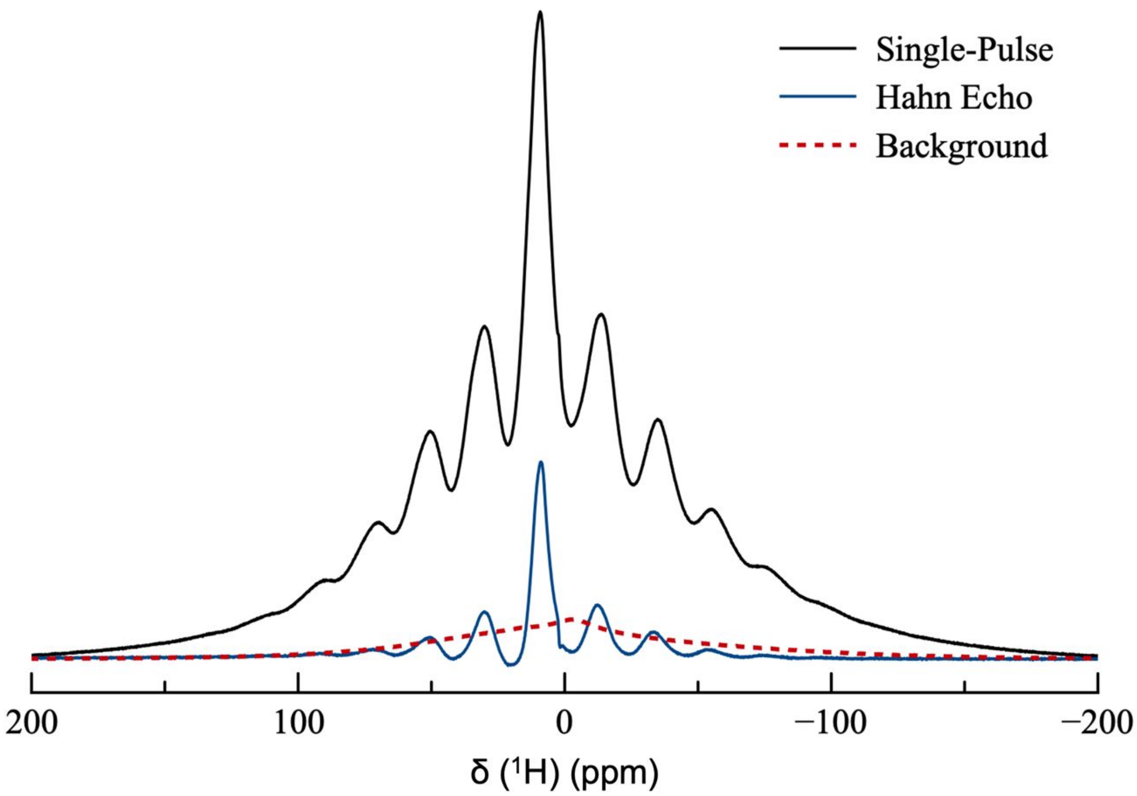

Fig. S6. ^{1}H MAS NMR spectra of $^{13}C/^{15}N$-labeled Tyr·HCl recorded at a field strength of 9.4 T. Spectra of Tyr·HCl were recorded at a spinning frequency of 8.0 kHz using direct excitation (black line) and a Hahn Echo (blue line). For comparison, the ^{1}H background signal of the 3D-printed stator was also plotted (dashed line). The ^{1}H spectra are normalized for the same number of scans.
